# *SULT1A1* gene deletion in *BRCA2*-associated male breast cancer: a link between genes and environmental exposures?

**DOI:** 10.1111/jcmm.12043

**Published:** 2013-05-27

**Authors:** Domenico Palli, Piera Rizzolo, Ines Zanna, Valentina Silvestri, Calogero Saieva, Mario Falchetti, Anna Sara Navazio, Veronica Graziano, Giovanna Masala, Simonetta Bianchi, Antonio Russo, Stefania Tommasi, Laura Ottini

**Affiliations:** a Molecular and Nutritional Epidemiology Unit, Cancer Research and Prevention Institute (ISPO)Florence, Italy; b Department of Molecular Medicine, “Sapienza” University of RomeRoma, Italy; c Division of Pathological Anatomy, Department of Medical and Surgical Critical Care, AOU Careggi, University of FlorenceFlorence, Italy; d Section of Medical Oncology, Department of Surgical and Oncological Sciences, University of PalermoPalermo, Italy; e Clinical Experimental Oncology Laboratory, National Cancer Centre of BariBari, Italy

**Keywords:** *SULT1A1*, copy number variations (CNVs), *BRCA2*, male breast cancer

## Abstract

SULT1A1, a member of sulfotransferase superfamily, is a drug and hormone metabolizing enzyme involved in the metabolism of a variety of potential mammary carcinogens of endogenous and exogenous origin. Interestingly, the metabolic activity of SULT1A1 can be affected by variations in gene copy number. Male Breast Cancer (MBC) is a rare disease and less investigated disease compared to female BC (FBC). As in FBC, the concurrent effects of genetic risk factors, particularly *BRCA2* mutations, increased exposure to estrogens and environmental carcinogens play a relevant role in MBC. By quantitative real-time PCR with TaqMan probes, we investigated the presence of *SULT1A1* gene copy number variations (CNVs) in a series of 72 MBCs. *SULT1A1* gene deletion was observed in 10 of the 72 MBCs (13.9%). In a multivariate analysis association between *BRCA2* mutation and *SULT1A1* gene deletion emerged (*p* = 0.0005). Based on the evidence that the level of SULT1A1 enzyme activity is correlated with CNV, our data suggest that in male breast tumors SULT1A1 activity may be decreased. Thus, it can be hypothesized that in a proportion of MBCs, particularly in *BRCA2*-associated MBCs, the level of estrogens and environmental carcinogens exposure might be increased suggesting a link between gene and environmental exposure in the pathogenesis of MBC.

SULT1A1, a member of sulfotransferase superfamily, is a drug and hormone metabolizing enzyme involved in the metabolism of a variety of potential mammary carcinogens of endogenous and exogenous origin, including oestrogens and polycyclic aromatic hydrocarbons (PAHs) 1.

Gene copy number variations (CNVs) are increasingly recognized to play a relevant role in the expression of drug metabolizing genes and in their respective enzymatic activities. In particular, the metabolic activity of SULT1A1 can be affected by variations in gene copy number 2.

Male breast cancer (MBC) is a rare disease, compared with female BC. The concurrent effects of genetic risk factors, particularly *BRCA2* mutations, increased exposure to oestrogens and environmental carcinogens play a relevant role in MBC 3. MBC is unaffected by the strong confounding effects of high disease frequency and of reproduction-related variables, thus, the complex effects of genetic, hormonal and environmental factors, involved in the pathogenesis of BC in both genders, can be better investigated in MBC.

On the basis of SULT1A1 biochemical properties, we investigated the presence of *SULT1A1* CNVs in a series of 72 MBCs characterized for relevant clinical-pathologic features, including *BRCA1/2* mutation status 4. *SULT1A1* CNVs were analysed by quantitative real-time PCR with TaqMan probes (Life Technologies, Carlsbad, CA, USA) comparing DNAs from tumour and blood from each MBCs included in the study. Normal breast tissue was used as calibrator sample. The fold change in studied gene copy number, normalized to endogenous control, was calculated using Relative Quantity (RQ) = 2^−ΔΔCt^.

*SULT1A1* gene copy number differences were found to occur in tumour compared with matched blood samples in 10 of the 72 MBCs (13.9%). In particular, *SULT1A1* gene deletion (RQ = 0.5) was observed indicating the presence of a single copy of *SULT1A1* gene in the 10 tumour samples compared with two copies (RQ = 1) detected in the corresponding blood samples. The results of blood and normal breast tissue paired samples were comparable in each patient.

As shown in Table 1, statistically significant association emerged between *SULT1A1* gene deletion and *BRCA2* mutations (*P* < 0.0001) and ER-negative status (*P* = 0.03). However, in a multivariate analysis only the association for *BRCA2* status persisted (*P* = 0.0005).

**Table 1:** Distribution of 72 MBC cases according to *SULT1A1* gene deletion and clinical-pathologic features

*SULT1A1*			
Parameter*	Deletion (%)	No deletion (%)	*P* value†
Family history of breast/ovarian cancer			
Negative	4 (8.2)	45 (91.8)	0.06
Positive	6 (27.3)	16 (72.7)	
Personal history of cancer			
Negative	7 (11.7)	53 (88.3)	0.18
Positive	3 (27.3)	8 (72.7)	
*BRCA1* status			
*BRCA1* wt	10 (14.5)	59 (85.5)	
*BRCA1* mutation	0 (0)	2 (100.0)	1.0
*BRCA2* status			
*BRCA2* wt	4 (6.3)	60 (93.7)	<0.0001
*BRCA2* mutation	6 (85.7)	1 (14.3)	
ER			
Negative	4 (40)	6 (60.0)	0.03^3^
Positive	6 (9.7)	56 (90.3)	
PR			
Negative	4 (25.0)	12 (75.0)	0.12
Positive	6 (10.7)	50 (89.3)	
HER2			
Negative	5 (9.4)	48 (90.6)	0.12
Positive	4 (22.2)	14 (77.8)	
Ki-67			
Low	4 (10.0)	36 (90.0)	0.2
High	5 (16.1)	26 (83.9)	
Histological grade			
G1/G2	3 (6.8)	41 (93.2)	0.06
G3	6 (25.0)	18 (75.0)	
Lymph node status			
Negative	1 (3.9)	25 (96.2)	0.35
Positive	4 (14.3)	24 (85.7)	
Total	10 (13.9)	62 (86.1)	

* Some data for each parameter are not available.

† From Fisher exact test.

‡ This association was not evident in a multivariate analysis.

To date, there are no data on CNVs of *SULT1A1* gene in BC. We found that a quite relevant proportion of MBCs (about 14%) showed *SULT1A1* gene deletion and, interestingly, that the deletion was significantly found in *BRCA2*-associated tumours. Based on the evidence that the level of SULT1A1 enzyme activity is correlated with CNV 2, our data suggest that in male breast tumours SULT1A1 activity may be decreased.

It has been reported that oestrogen sulfotransferases are frequently decreased in breast carcinomas and this may result in an increased exposure of mammary tissue to oestrogens 5. Very recently, SULT1A1 has been shown to play an important role in the detoxication of PAHs in lung cells 6. Thus, based on our results, it can be suggested that in a proportion of MBCs, particularly in *BRCA2*-associated MBCs, the level of oestrogens and environmental carcinogens exposure might be increased. This could be particular relevant considering the important role of oestrogens in MBC pathogenesis and the molecular crosstalk between oestrogens and *BRCA2* gene 7. Intriguingly, we have previously shown an interaction between *BRCA* carrier status and occupational exposure to chemicals, such as PAHs, in MBC patients 8. Thus, our present results may help to clarify possible pathogenetic mechanisms underlying this interaction.

Overall, our data suggest that MBCs, particularly *BRCA2*-associated MBCs, may be characterized by low SULT1A1A enzymatic activity thus suggesting a link between gene and environmental exposure and opening interesting questions on clinical settings.
